# The Utility of Study Insight Analytics (SIA) Algorithms in Identifying Scoring Error Risks in the Clinical Dementia Rating scale (CDR)

**DOI:** 10.1002/alz.085766

**Published:** 2025-01-03

**Authors:** Selam Negash, Barbara Echevarria, Raymond Blattner, Christopher Randolph

**Affiliations:** ^1^ WCG Clinical Endpoint Solutions, Princeton, NJ USA

## Abstract

**Background:**

Clinical trials in Alzheimer’s Disease (AD) suffer from high failure rates, in part due to imprecision in endpoint measurements that introduces noise^1^. SIA is a quantitative approach that utilizes algorithms to identify inconsistencies in measurements that may be indicative of problematic scale administration and/or scoring errors. The CDR, a sole primary and key secondary endpoint in many AD trials, can be challenging to score, particularly in early symptomatic and mild diseases^2^. The goal of this study was to develop and validate SIA algorithms for CDR, and to evaluate their incidence and association with scoring errors.

**Methods:**

Aggregated data from 40,148 CDR reviewed assessments across 34 multinational trials of pre‐clinical, early symptomatic, and mild to moderate dementia due to AD were analyzed. Algorithms indicative of scoring errors on the basis of clinical judgement were developed. These were then subjected to a validation procedure to determine both the rates flag trigger, and the degree to which each flag was associated with increased scoring errors. The development and validation of one of these flags (the cognitive‐functional difference score)^3^ is described in detail as an example. Algorithms that fire relatively infrequently (to minimize false positives), independently (indicating increased probability of scoring errors), and associated with increased error rates were identified.

**Results:**

Five flags emerged from the validation process to meet criteria of relatively low firing rate (<10%), association with increased rates of scoring errors, and having relatively orthogonal relationships with each other. The latter goal was established by a strong linear relationship between number of flags triggered and scoring error rate, such that as the number of flags triggered increased, the mean error rate was significantly elevated (estimate = 0.20, SE = 0.008; p < 0.0001).

**Conclusion:**

The present study, using a large, aggregated dataset across multiple AD trials, demonstrated the utility of SIA algorithms in detecting data inconsistencies that help identify problematic assessments in CDR. The very strong association between flags triggered and error rates suggests that the algorithms can serve as a proxy for identifying assessments that are predictive of scoring errors and to surface these assessments for review/remediation.

